# Stand When You Can: development and pilot testing of an intervention to reduce sedentary time in assisted living

**DOI:** 10.1186/s12877-020-01647-z

**Published:** 2020-08-06

**Authors:** M. L. Voss, J. P. Pope, R. Larouche, J. L. Copeland

**Affiliations:** 1 Department of Kinesiology & Physical Education, University of Lethbridge, 4401 University Drive, Lethbridge, AB T1K 3M4 Canada; 2 Faculty of Health Sciences, University of Lethbridge, Lethbridge, Canada

**Keywords:** Aging, Assisted living, Sedentary behaviour, Physical function, Social ecological model

## Abstract

**Background:**

Prolonged daily sedentary time is associated with increased risk of cardiometabolic diseases, impaired physical function, and mortality. Older adults are more sedentary than any other age group and those in assisted living residences accumulate more sedentary time as they often have little need to engage in light-intensity or standing activities such as cleaning or meal preparation. This “low movement” environment can hasten functional decline. Thus, the purpose of this study was to develop a multi-level intervention to reduce and interrupt sedentary time within assisted living residences and conduct a pilot study to determine if the intervention is feasible and if further testing is warranted.

**Methods:**

“Stand When You Can” (SWYC) was developed using a Social Ecological framework based on a review of literature and consultation with residents and staff at assisted living residences. After development, a six-week pilot study was conducted in two different residences with 10 older adults (82.2 ± 8.7 years). Before and after the 6 weeks, ActivPAL™ inclinometers were used to measure daily movement behaviours and self-report questionnaires assessed time spent in different sedentary behaviours and quality of life. Physical function was assessed using the Short Physical Performance Battery. Paired sample t-tests examined pre-post differences for pooled data and individual sites. At the end of the pilot study, feedback on the intervention was gathered from both residents and staff to examine feasibility.

**Results:**

There was a trend towards a decrease in self-reported sitting time (142 min/day; *p* = 0.09), although device-measured sedentary time did not change significantly. Participants with lower physical function at baseline showed clinically meaningful improvements in physical function after the 6 weeks (*p* = 0.04, *Cohen’s d* = 0.89). There was no change in quality of life. Residents and staff reported that the intervention strategies were acceptable and practical.

**Conclusion:**

This study suggests that a multi-level intervention for reducing prolonged sedentary time is feasible for implementation at assisted living residences. The intervention could potentially help delay functional decline among older adults when they transition to a supportive living environment. Longer and larger trials to test the efficacy of SWYC are necessary.

**Trial registration:**

Name of Clinical Trial Registry: clinicaltrials.gov

Trial Registration number: NCT04458896.

Date of registration: July 8, 2020. (Retrospectively registered).

## Background

The global population is aging with an estimated 2 billion older adults (≥65 years) worldwide in the next 30 years [1]. These demographic changes present unique challenges for promoting health and well-being to older adults. Due to an increase in life expectancy, the number of older adults experiencing chronic conditions and functional limitations has increased, resulting in greater demand for alternative housing options. The World Health Organization [2] forecasts the demand for residential care to quadruple globally by 2050. Supportive or assisted living residences allow older adults to maintain independence but have access to assistance with some activities of daily living and opportunities for social interaction. There is no standard definition of assisted living and it can include a range of services; most have at minimum 24-h emergency assistance, meal service, and housekeeping support. With these supports in place, older adults who transition to assisted-living often have little need to engage in light-intensity or standing activities such as household chores or meal preparation. As a result, older adults in assisted living accumulate more sitting time and less time standing and walking compared to their peers who live independently [3–5].

Regular physical activity has well-known benefits in mitigating chronic disease and promoting healthy aging. Unfortunately, people tend to become less active and more sedentary with age. Older Canadians have been shown to accumulate only 14.5 min of moderate to vigorous physical activity (MVPA) per day on average [6] and 90% of older Canadians accumulate more than 8 h of sedentary time per day [7]. Sedentary time refers to activities completed in a seated or reclined position with minimal energy expenditure while awake [8], and excessive time spent sedentary is associated with a number of negative health consequences among older adults [9]. Regular physical activity can attenuate the effects of sedentary time [10, 11], and even a small volume of activity can reduce mortality among those with chronic disease [12]. However, very few older adults meet even the minimum guidelines for physical activity [6]. The combination of low amounts of physical activity and high volumes of sedentary time can be especially detrimental to health as well as physical and cognitive function [9, 10]. Reducing sedentary behaviour, something older adults are engaging in for the majority of their waking hours, could be beneficial, especially for individuals who are uninterested, or perhaps unable, to participate in regular, purposeful exercise.

Reducing or regularly interrupting sedentary time has been associated with a lower risk of chronic disease and better physical function [13, 14]. Data from the National Health and Nutrition Examination Survey demonstrate that more breaks in sedentary time are associated with a reduced risk of metabolic syndrome, lower waist circumference, body mass index (BMI), and blood triglycerides [13, 15]. Breaks in sedentary time have also been associated with better physical function, self-rated health, and mental health [16]. Sardinha et al. [17] found that older adults with > 7 breaks per hour of sedentary time were less likely to require assistance or be unable to complete various activities of daily living compared to those with ≤7 breaks per hour.

With evidence demonstrating potential benefits from limiting sedentary time, intervention research is rapidly developing. Most studies with older adults have typically sought to change sitting time by educating people about the detriments of sedentary time, increasing self-awareness of sitting behaviours, and facilitating goal setting to change behaviour [18–22]. Some of these interventions have been successful, with reductions in self-reported sedentary time between 76 and 132 min per day [19, 20]. Studies reporting device-measured sedentary time have reported smaller decreases (~ 25 min per day) [18, 21, 22]. Gardiner et al. [21] also noted a 4% increase in sit to stand transitions with an individual behaviour change intervention. Although these findings are promising, most of these studies were conducted with community-dwelling older adults who were relatively young, healthy, and active. These findings may not directly translate to those in assisted living, as they are often more sedentary and less physically active by comparison. There is a need for more research in assisted living and other residential care environments [23].

The Social Ecological Model serves as a useful framework to guide the development of an intervention targeting sedentary time among older adults living in assisted living residences. The Social Ecological Model posits that behaviours have multiple levels of influence: *individual* attributes and choices, the *social* environment, the *physical* environment, and *organizational/policy* factors [24]. Stokols [24] suggested that health promotion interventions should integrate person-focused and environment-focused strategies. Further, because sedentary behaviour is ubiquitous across leisure, transport, and household domains [25], the physical, organizational, and social environment must support and enable individual strategies to produce meaningful and long-term change in older adults' sitting behaviours.

Pilot studies and feasibility trials are recommended when there is little research in a given subject area or data are lacking for a particular intervention technique, as is the case with sedentary behaviour in older adults in assisted living [26]. The purpose of this study was to develop a multi-level intervention to reduce sedentary time among residents in assisted living and conduct a pilot study to examine the feasibility of the intervention and determine if further testing is warranted. The specific objectives were 1) to examine the acceptability and practicality of the intervention; and 2) conduct preliminary efficacy testing of the intervention through a 6-week pilot trial.

## Methods

### Intervention development

Stand When You Can (SWYC) was developed in three steps: 1) review of the literature; 2) discussions with residents in assisted living; 3) consultation with staff at assisted living residences. The procedures for steps two and three were reviewed and approved by the University of Alberta Health Research Ethics Board, protocol number 00075411.

The review of literature showed that interventions targeting increased physical activity are not effective for reducing sedentary time, and it is important to specifically focus on sedentary behaviour [27, 28]. Interventions in community-dwelling older adults have primarily focused on individual behaviour change including education and goal setting [20, 21], while some studies in nursing homes have focused on educating staff about the benefits of reducing sedentary time among residents [29, 30]. Since both strategies have been successful at reducing sedentary time and slowing declines in physical function, they were referenced as a starting point for SWYC. Due to the limited research in assisted living dwellings, interventions in an office workplace context were also examined. Assisted living shares some similarities with workplaces in that behaviour is influenced by the social and physical environment, especially in common areas, but individuals have autonomy over their own behaviours and own private spaces (e.g.: their suites). Preliminary evidence suggests that multi-level interventions to reduce sedentary time are more successful in workplaces than interventions that target only one level of influence [31]. Thus, some strategies from these interventions (e.g. educating managers and policymakers, using point of decision prompts to encourage behaviour change in public areas, and including furniture that promotes standing instead of sitting) were adapted for SWYC.

After the review of literature, we conducted focus groups with older adults in assisted living to identify barriers and motivators to reducing sedentary time. Full details of the focus groups are presented elsewhere [32], but the key findings that informed our intervention are summarized here. In general, residents identified a negative connotation associated with the term “sedentary”, and a perception that only passive or solo seated behaviours (e.g. watching TV alone) were truly sedentary. Sedentary behaviours that were cognitively engaging, such as reading or playing games, were believed to be associated with health benefits, which is consistent with other research with community dwelling older adults [33, 34]. These findings were informative in shaping the education material and how information was communicated, with emphasis placed on breaking up prolonged periods of sitting, rather than “avoiding” seated activities that older adults enjoy and believe are beneficial.

In terms of barriers and motivators to reduce sedentary time, residents identified many individual factors that influence their sedentary time. For example, avoiding discomfort and maintaining mobility were important motivators to reduce sedentary time, while lack of motivation, fatigue, pain, and fear of falling were all discussed as barriers. Social and organizational factors also emerged as important considerations. Residents described social norms and a lack of activities in the evening and on weekends that led them to remain in their respective suites engaged in more passive sedentary activities. Conversely, social engagement, encouragement from others, and opportunities to engage in interesting activities were common motivators to reduce sedentary time. We concluded that intervention strategies should target a shift in habits at the individual, social, and organizational levels, with strategies that provide education and encouragement, increase motivation, and offer interesting and enjoyable alternatives to prolonged periods of passive sitting.

Following the literature review and focus groups, a list of preliminary strategies was developed. Staff were then consulted to gather feedback about the feasibility of the proposed strategies. A total of 16 staff (all women) from 5 different ALRs were interviewed, including managers, activity coordinators (AC), and kitchen and housekeeping personnel. Overall, staff felt most of the strategies we presented were feasible. One proposed tactic that was deemed problematic was encouraging people to change seats during mealtimes or organized activities; staff indicated there are group social dynamics at play in ALRs that could make that approach difficult to implement. They also suggested that changing furniture to ensure all chairs had arm rests to help people stand more easily was an issue due to space and logistics. Both of these strategies were removed from SWYC.

It was clear from resident discussions and staff consultation that each facility is unique and thus SWYC would need to be flexible with several different components that could be implemented in tandem or individually. Such a flexible and adaptable intervention approach has been used successfully by Buman et al. [35] in a workplace trial to reduce sedentary behaviour. The resulting collection of strategies target different levels of behavioral influence to encourage less sitting time (see Additional file 1, Table S1).

### Pilot trial

#### Participant Recruitment

An invitation letter was sent to a local foundation in a small city in Western Canada that has 13 ALRs that operate on a government-subsidized funding model. The managers and activity coordinators (ACs) of two different residences agreed to participate (Site A (47 residents) and Site B (115 residents)). Both residences provide the same level of care and offered meals, housekeeping, and social activities within the facility; residents are required to be independently mobile with or without a walking aid. Additional medical care is accessed through community-based clinics or home care and no medical staff are present at the residences.

**Fig. 1:**
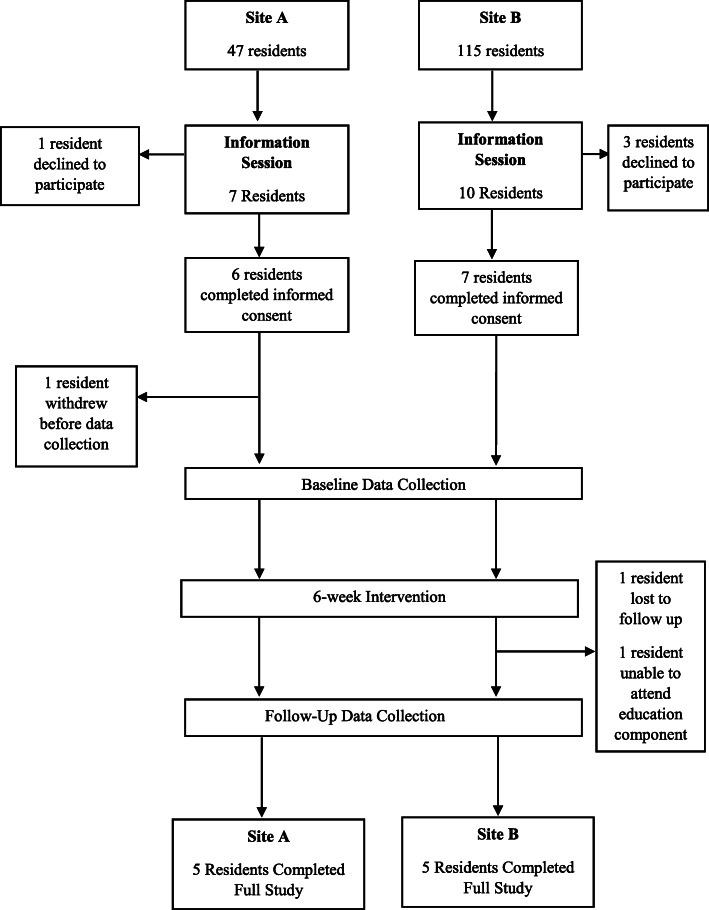
Flow diagram showing participant recruitment and enrollment and study timeline

A meeting was held with management at the residences to provide an overview of the intervention, discuss the support needed from the ACs, and schedule study sessions. An information recruitment session was held at both sites to provide interested residents with information about the study (Fig. 1). Overall, 17 residents attended the information sessions between the two residences (Site A *n* = 7; Site B *n* = 10). Thirteen residents signed up for the study and provided informed consent, with one withdrawing from the study before baseline data collection, leaving a final sample of 12 (91% female, 82.67 ± 7.98 years) at the beginning of the study. Due to limited research in this setting, we were unable to predict recruitment rates or complete a power calculation, therefore, a priori sample size estimates were not calculated.

##### Study design

Before the intervention, assessments were conducted over two sessions. At the first session, participants completed a questionnaire package to collect demographic data, self-reported sedentary time, and quality of life. Participants were fitted with an activPAL™ inclinometer (PAL Technologies, Glasgow, Scotland) and 1 week later the activPAL™ was collected and participants completed assessments of physical function. The SWYC intervention strategies were then implemented for 6 weeks. The full SWYC program was envisioned as a menu from which ALRs could select strategies that were most appropriate for their specific residence; those implemented in the pilot trial are noted with an asterisk in Table S1 (Additional file 1). After the 6-week trial, all assessments were repeated. All study sessions took place at the respective residences to mitigate transportation barriers. Study procedures for the pilot trial were approved by the University of Lethbridge Human Participant Research Committee, Protocol #2019–001 and all participants provided written informed consent. The pilot trial was retrospectively registered at clinicaltrials.gov (NCT04458896) July 8, 2020.

#### Efficacy measures

##### Quality of life

Quality of life was measured with the EQ-5D-5L and the ICEpop CAPability Measure for Older People (ICECAP-O). We used both tools because they measure distinctly different aspects of quality of life. The EQ-5D-5L is a generic measure of health status that assesses mobility, self-care, usual activities, pain/discomfort, and anxiety/depression, along a five-point scale (no problems, slight problems, moderate, severe, and unable to do the action/extreme) [36]. The EQ-5D-5L generates a health state that was translated to a summary index value using the Crosswalk Value Index Calculator (US values, euroqol.org). It also includes a visual analogue scale for overall health anchored between 0 (worst health imaginable) to 100 (best health imaginable) [36]. The EQ-5D-5L has a discriminatory power of 0.68 and a test-retest reliability of 0.69 [37].

The ICECAP-O is a broader measure of quality of life that does not focus on physical health but covers the domains of attachment (love and friendship), security (thinking about the future without concern), role (doing things that make you valued), enjoyment (enjoyment and pleasure), and control (independence). Scores for the ICECAP-O range from 0 (no capability) to 1 (maximum capability). The ICECAP-O has been shown to be reliable (intra-class correlation coefficient = 0.80) and have good construct validity as a measure of quality of life [38, 39].

##### Sedentary time

Sedentary time was assessed with the activPAL4™ inclinometer which measures movement patterns 24 h/ per day and can monitor body positions, which makes them useful for measuring sedentary time. activPALs have been found to be valid and reliable in comparison to direct observation (*R*^*2*^ = 0.94) [40]. The activPAL4s were waterproofed using a nitrile sleeve and affixed to the thigh using Tegaderm (3 M Medical, USA).

While self-report tools tend to underestimate sedentary time, they can provide information about domain-specific sedentary time [41]. The Longitudinal Aging Study Amsterdam (LASA) Sedentary Behaviour Questionnaire estimates self-reported sedentary time by asking participants about time (hours:minutes) spent in 10 sitting behaviours on an average weekday or weekend day [41]. The questionnaire has a test-retest reliability of 0.71 (95% CI 0.57–0.81), but may underestimate total sedentary time by as much as 2.1 h [41]. Visser and Koster [41] found the six domains of napping, reading, listening to music, watching TV, engaging in seated hobbies, and talking to friends had the highest correlation with device-measured sedentary time. Thus, we only included these six domains when calculating self-reported sedentary time.

##### Physical function

Prior to the physical assessments, participants completed the Get Active Questionnaire [42] and resting heart rate and blood pressure were taken with an automated blood pressure cuff (UA-787, Life Source A&D Medical, Mississauga, Ontario) to ensure it was safe for them to complete the protocol. Height and weight were also measured using a stadiometer (Seca, Hamburg, Germany) and scale (Seca, Hamburg, Germany). Grip strength was measured using a dynamometer (Creative Health Products Inc., USA) following CSEP testing guidelines [43].

The Short Physical Performance Battery (SPPB) is an objective assessment tool for evaluating lower extremity functioning in older adults and it includes assessments of gait speed (4 m walk), standing balance (side by side, semi-tandem, and tandem), and 5 timed chair rises [44]. The SPPB has been shown to have good predictive and concurrent validity and reliability (intraclass correlation coefficient > 0.70) in measuring physical function in older adults [45] and is also predictive of mobility impairment [46]. A change of 0.5 is considered to be a small meaningful change in physical function while a 1-point change considered a substantial meaningful change [47]. The entire battery of tests was conducted in a circuit format that took approximately 20 min to complete.

##### Post-intervention feedback

We collected feedback forms from residents after the 6-week intervention to gain an understanding of which strategies they used most frequently and how much they liked or disliked the different components of the intervention. We also interviewed the ACs to receive staff feedback about ways we could improve the program for future iterations. Feedback questions included what components seemed to resonate with residents, which components were not utilized, and any other strategies that we could leverage to improve delivery and support. All staff provided written informed consent prior to the interview.

### Analysis

The activPAL™ results were analysed using the CREA – beta algorithm in the PALbatch software (v8.10.6.33, PAL Technologies, Glasgow, Scotland). This algorithm considers 24 h wear time and classifies lying time as primary (i.e. during the night) or secondary (i.e. during the day). Both primary and secondary lying time were excluded from consideration for “sedentary time” as it is unknown whether the participants were asleep during these periods or simply reclining. All statistical analyses were completed in SPSS Statistics 24 (v 24.0.0.1 IBM) and missing values were not replaced. Baseline differences between sites were examined using independent sample t-tests; paired-sample t-tests were used to compare changes over the 6-week intervention. Pooled data were analyzed and although sample sizes were small, given the pilot nature of the study, analyses were also conducted separately for each site.

## Results

Twelve participants started the study; one participant was lost to follow up due to medical complications unrelated to the study and another participant was unable to attend the education session and one session of baseline data collection, and therefore was excluded from the analysis. This left 10 participants with complete data for analysis (Fig. 1). The data analysed are available from the corresponding author on reasonable request.

### Baseline differences

**Fig. 2:**
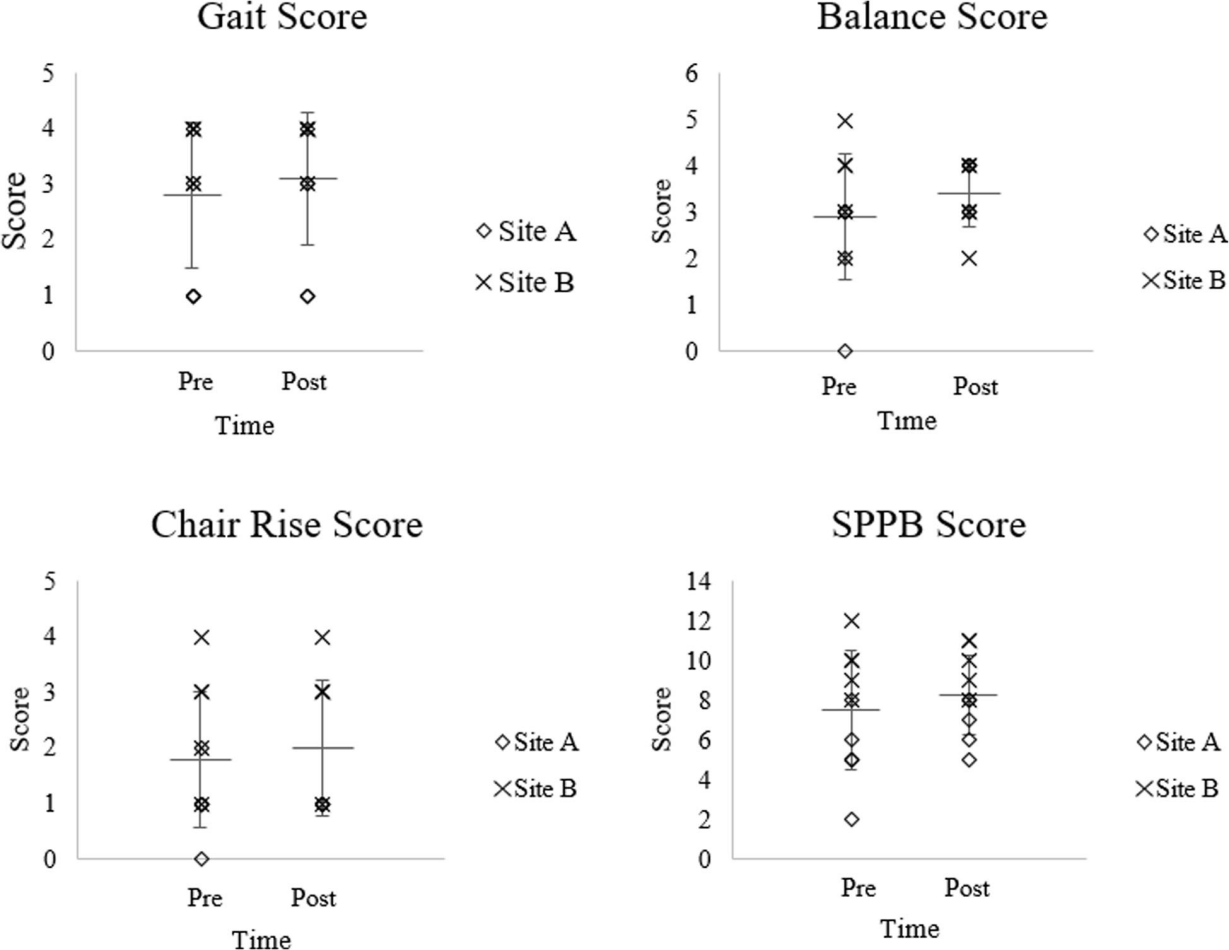
Pre- and post-intervention measures of physical function; lines represent overall means ± standard deviation, markers represent individual data points

Table 1 shows the participants’ characteristics by site. There was a significant difference in age with participants at Site B being significantly older, on average (Site A: 76.20 ± 8.32, Site B: 88.20 ± 3.49, *t* = − 2.97, *p* = 0.02) (Table 1). There were no differences in movement patterns between sites, although significant differences existed in total SPPB scores (Site A: 5.20 ± 2.17, Site B: 9.80 ± 1.48, *t* = 3.92, *p* < 0.01) due to slower gait speed (Site A: 0.73 ± 0.26, Site B: 0.89 ± 0.14, *t* = − 2.36, *p* = 0.05) and chair rises (Site A: 34.15 ± 17.77, Site B: 14.05 ± 35, *t* = − 2.67, *p* = 0.03) among participants at Site A (Fig. 2). Significant differences also existed for self-reported quality of life (ICECAP-O) (*t* = − 3.38, *p* = 0.01), with Site B having higher scores, but there were no significant differences in self-reported sedentary time or EQ-5D scores.

**Table 1:** Baseline characteristics

Variable	Site A	Site B	Difference between sites t (p)	Overall
N (%female)	5 (80%)	5 (100%)	–	10 (90%)
Age	76.2 ± 8.3	88.2 ± 3.5	−2.97 (0.02)*	82.2 ± 8.7
Years in facility	3.1 ± 4.0	2.9 ± 2.1	0.11 (0.92)	3.0 ± 3.0
Marital status			2.5 (0.29)^a^	
Widowed	3 (60%)	5 (100%)		8 (80%)
Divorced	1 (20%)	0		1 (10%)
Never married	1 (20%)	0		1 (10%)
BMI	31.0 ± 5.3	29.7 ± 7.10	0.31 (0.77)	30.4 ± 6.0
LASA – weekday (hours per day)	14.5 ± 4.6	14.7 ± 3.7	−0.05 (0.96)	14.6 ± 3.9
LASA – weekend (hours per day)	10.3 ± 3.61	11.5 ± 4.7	− 0.44 (0.68)	11.0 ± 4.0
EQ-5D health index	0.64 ± 0.21	0.84 ± 0.09	−1.95 (0.09)	0.74 ± 0.20
EQ-5D health today	70.0 ± 12.7	85.0 ± 11.2	− 1.98 (0.08)	77.5 ± 13.8
ICECAP-O	0.74 ± 0.10	0.92 ± 0.06	−3.38 (0.01)*	0.83 ± 0.12
SPPB score	5.2 ± 2.2	9.8 ± 1.5	−3.92 (0.004)*	
Sitting time (minutes/day)	614.6 ± 132.3	578.4 ± 237.7	0.28 (0.77)	596.5 ± 182.4
Minutes in seated bouts > 30 min	363.9 ± 144.1	388.3 ± 204.0	−0.22 (0.83)	376.1 ± 167.0
Sit to stand transitions per day	49.2 ± 10.43	41.0 ± 5.7	1.5 (0.16)	45.1 ± 9.0
Steps per day	5762 ± 5788	4586 ± 1273	0.44 (0.67)	5173 ± 3999

All values expressed as mean ± standard deviation

*SPPB* Short Physical Performance Battery, *LASA SBQ* Longitudinal Aging Study Amsterdam Sedentary Behaviour Questionnaire, *ICECAP – O* IcePOP CAPability measure for Older People

^a^χ^2^ test conducted to compare means for marital status

* *p* < 0.05

### Pre- and post- intervention changes

There were no significant changes in device-measured total sedentary time, standing time, average steps, sit to stand transitions, or number of bouts of sitting > 30 min (Table 2). Device-measured movement patterns by weekday and weekend day are shown in Additional file 3, Table S3. At Site A, participants had significantly more sit-to-stand transitions at baseline on weekdays compared to weekends (Weekdays: 51.35 ± 10.76, Weekends: 44.40 ± 10.00, *t* = 3.38, *p* = 0.03).

**Table 2:** Device-measured movement variables pre- and post-intervention

Variable	Time	Site A		Site B		Overall		
		Minutes per day	Cohen’s ***d***	Minutes per day	Cohen’s ***d***	Minutes per day	t (p)	Cohen’s ***d***
Sitting time	Pre	614.6 ± 132.3	0.25	578.4 ± 237.7	0.10	596.5 ± 182.4	0.31 (0.75)	0.04
	Post	577.5 ± 158.0		602.3 ± 232.8		589.9 ± 188.0		
Stepping time	Pre	70.5 ± 54.1	0.16	63.7 ± 18.9	0.16	67.1 ± 38.4	− 0.91 (0.39)	0.16
	Post	93.1 ± 84.8		57.2 ± 13.7		75.1 ± 60.3		
Standing time	Pre	186.5 ± 81.8	0.15	242.6 ± 163.4	0.30	214.5 ± 125.4	0.54 (0.60)	0.11
	Post	197.9 ± 67.5		207.3 ± 108.9		202.6 ± 85.6		
Upright time	Pre	257.0 ± 127.3	0.25	306.2 ± 162.6	0.25	281.6 ± 140.1	0.15 (0.88)	0.03
	Post	290.9 ± 142.1		264.5 ± 110.5		277.7 ± 120.8		
Sitting time in bouts > 30 min	Pre	364.0 ± 144.1	0.25	388.3 ± 204.0	0.13	376.1 ± 167.0	0.29 (0.78)	0.05
	Post	320.4 ± 193.1		414.0 ± 185.8		367.2 ± 185.4		
Steps per Day	Pre	5761.6 ± 5788.5	0.27	4584.8 ± 1273.1	0.78^a^	5173.2 ± 3999.6	−0.94 (0.37)	0.20
	Post	7840.0 ± 9032.1		4083.8 ± 756.9		5962.9 ± 6358.5		
Sit to Stand Transitions per Day	Pre	49.2 ± 10.4	0.60^a^	41.0 ± 5.7	0.28	45.1 ± 9.0	−0.72 (0.49)	0.15
	Post	54.60 ± 7.09		39.4 ± 5.8		47.0 ± 10.1		

All values expressed as mean ± standard deviation; All times are expressed as minutes per day

^a^moderate effect size

One participant was excluded from analysis of self-reported sedentary time due to invalid data (sum of values exceeded 24 h) leaving *n* = 9. Across the six domains of napping, seated reading, listening to music, watching TV, seated hobbies, and talking to friends, there was a large but non-significant 142.2 min/day decrease in weekday sedentary time (*t* = 1.96, *p* = 0.09, *Cohen’s d* = 0.90) (Table 3). Self-report data from each domain of the LASA questionnaire can be viewed in Additional file 2, Table S2.

**Table 3:** Self-reported sedentary time pre- and post-intervention

Variable	Time	Site A		Site B		Overall	
		Weekday	Weekend	Weekday	Weekend	Weekday	Weekend
LASA – all domains	Pre	871.25 ± 278.04	616.25 ± 216.45	879.00 ± 222.38	690.60 ± 280.55	875.56 ± 231.81	657.55 ± 241.78
	Post	697.50 ± 209.82	756.30 ± 315.28	717.00 ± 267.57	588.60 ± 251.57	708.34 ± 228.94	663.13 ± 277.00
	Cohen’s *d*	0.71^¥^	0.51^¥^	0.68^¥^	0.38	0.73^¥^	0.05
LASA – 6 domains	Pre	616.25 ± 203.61	436.25 ± 143.02	627.00 ± 188.67	537.00 ± 258.16	622.22 ± 182.69	492.00 ± 209.31
	Post	480.00 ± 146.97	415.05 ± 249.95	480.00 ± 129.03	393.60 ± 140.94	480.00 ± 128.16	403.72 ± 182.99
	Cohen’s *d*	0.77^¥^	0.10	0.91^§^	0.69^¥^	0.90^§^	0.46

All values expressed as minutes per day (means ± standard deviations); LASA – 6 domains: sum of napping, reading, music, watching TV, seated hobbies, and talking to friends

^¥^moderate effect size; ^§^large effect size

Overall, there was no significant change in quality of life as assessed by either the EQ-5D or the ICECAP-O questionnaires over the 6 weeks (Table 4). Pooled analysis showed there was a trend towards improvement in total SPPB score (Fig. 2), although this did not reach significance (*p* = 0.09, *Cohen’s d* = 0.18). Participants at Site A demonstrated statistically and clinically significant changes in physical function after the 6 weeks (Fig. 2) with improvements in tandem balance time (Pre: 5.08 ± 2.62, Post: 8.54 ± 2.19, *t* = − 2.97, *p* = 0.04, *Cohen’s d* = 1.43) and total SPPB score (Pre: 5.20 ± 2.17, Post: 6.80 ± 1.30, *t* = − 3.14, *p* = 0.04, *Cohen’s d* = 0.89). There were no significant changes in physical function at Site B, although changes in some measures approached moderate effect sizes.

**Table 4:** Quality of life scores pre- and post-intervention

Variable	Time	Site A		Site B		Overall		
		Value	Cohen’s ***d***	Value	Cohen’s ***d***	Value	t (p)	Cohen’s ***d***
ICECAP - O	Pre	0.74 ± 0.10	0.14	0.92 ± 0.06	0.42	0.82 ± 0.12	0.99 (0.35)	0.14
	Post	0.72 ± 0.17		0.89 ± 0.08		0.80 ± 0.15		
EQ-5D Health Index	Pre	0.64 ± 0.21	0.06	0.84 ± 0.09	0.44	0.74 ± 0.19	0.39 (0.70)	0.06
	Post	0.65 ± 0.14		0.81 ± 0.03		0.73 ± 0.13		
EQ-5D Health Today	Pre	70.0 ± 12.8	0.43	85.0 ± 11.8	0.43	77.5 ± 13.8	−0.16 (0.88)	0.07
	Post	77.0 ± 19.2		80.0 ± 12.2		78.5 ± 15.3		

All values expressed as mean ± standard deviation

### Resident feedback

Over half of participants reported enjoying all intervention components, although the physical and social environment strategies did not score as high on likeability or usage as the individual behaviour change strategies. The participants indicated that they learned a lot and that they could adapt the strategies presented in the education session to match their own lifestyle. One participant reported they would prefer more check-ins and contact with staff throughout the program and another indicated they would have liked the program to last longer.

### Staff feedback

The ACs reported SWYC was easily implemented at both sites. They recommended that future iterations incorporate more self-regulation through logbooks or checklists. The scavenger hunt was extremely popular and well-received, and the standing table was used at both sites, although infrequently. It was suggested that an activity (e.g. puzzle or game) be placed at the table to encourage greater use. Both ACs indicated that they did not make any specific changes to the way in which they implemented regular programming to reduce or interrupt prolonged sitting.

## Discussion

SWYC is a novel intervention to reduce sedentary time in assisted living, designed through a participatory action framework to address four levels of the Social Ecological Model. It is a low-cost, flexible, intervention that does not require specialized staff or equipment, making it potentially scalable and adaptable to different types of residences. A 6-week pilot study showed that SWYC was acceptable to both residents and staff and was feasible in these residences. There was also preliminary evidence of effectiveness at Site A, despite the short duration and small sample size.

Pooled data indicated no significant changes in device-measured movement patterns. Self-reported sedentary time was similar to device-measured values at baseline and indicated a trend for reductions in both weekday (142 min/weekday) and weekend (89 min/weekend day) sitting, although this reduction was not observed in the device-measured data. The reductions in self-reported sitting time are similar to those reported by Maher et al. [20] after a 12-week intervention targeting individual behaviour change (132.6 min/day). The post-intervention reduction in device-measured sedentary time observed at Site A was consistent with findings in community-dwelling older adults that have reported reductions in sitting of approximately 30-min per day [18, 21, 22] and a decrease of this magnitude could have a meaningful impact on physical function and mobility [48, 49]. Furthermore, participants at Site A increased their daily sit to stand transitions which could also be beneficial. However, it is important to note there were no significant changes in device-measured sedentary time overall and participants at Site B actually increased their device-measured sitting time by 24 min a day (*p* = 0.537). These different trends in results between sites are difficult to explain given the short duration of the study and small sample size. The baseline differences in physical function and quality of life between the participants at the two sites could have influenced their motivation to reduce their sedentary time. Specifically, Site B participants had fewer functional limitations and better quality of life, thus the educational information aimed at promoting less sedentary behaviour may have been less effective or motivating for those people. It is also possible that the staff at the two sites approached the intervention strategies differently and this points to the need for assessments of intervention fidelity in future trials. These findings also highlight the importance of including device-based measurements as self-report tools are prone to recall and social desirability biases and possibly more so after providing education on the benefits of reducing sedentary time.

Evidence for the health effects of sedentary time interventions is sparse [9, 50], although observational studies suggest that even small reductions in sedentary time could be beneficial, especially in older adults with low physical activity and reduced mobility. For example, each 30-min increase in sedentary time was associated with a 17% increase in rates of major mobility disability in community-dwelling older adults [49]. Among older adults in assisted living, each additional hour of sedentary time was associated with slower 400 m walk times and a lower score on the SPPB [48]. Although we did not find significant changes in physical function overall, some trends in the results, specifically at Site A, were promising. Participants at Site A had a significantly lower SPPB score at baseline compared to Site B, so the small stimulus of increased sit to stand transitions may have been enough to improve lower extremity strength. Sardinha et al. [17] found breaks in sedentary time were positively associated with higher physical function and other studies have shown that increasing sit to stand transitions can prevent declines in physical function over 12-month follow up [29, 30]. Small changes in gait speed and SPPB scores can have meaningful effects on physical function and fall risk [51, 52]. A larger trial is needed to determine if the intervention strategies are more effective among older adults with lower physical function.

Stand When You Can was acceptable and feasible within this particular model of assisted living. The services offered, number and type of staff, and amount of social programming can vary widely across different assisted living facilities and different levels of residential care. While we cannot comment on how the intervention strategies would work in other residences, most of the strategies could be adapted and implemented in any communal living environment. The results of this pilot study suggest that better support and engagement at the social and organizational levels could enhance the effectiveness of SWYC. Previous studies have shown the benefit of including staff in interventions to reduce sedentary time in residential care [29, 30], and one of the organizational level strategies included in SWYC was for staff to encourage standing or moving breaks in all regular programming. However, the post-intervention interviews with the ACs revealed that their involvement was minimal, so future studies using SWYC should place a greater emphasis on staff education and engagement. The ACs were provided with a copy of the educational materials that were given to the residents and were provided with tips to increase stand and stretch breaks during group activities, however, educational sessions and material specifically for staff may be necessary to improve the delivery of the SWYC program. Including a questionnaire to measure self-reported sedentary time during the staff workshop may also serve to increase staff awareness of sitting behaviours and facilitate a discussion around the benefits and drawbacks of prolonged sitting and how that fits within the context of assisted living. Furthermore, the ACs suggested that increasing the focus on intervention strategies involving friends or family outside of the residence may also improve intervention effectiveness.

Strengths of this intervention include the participatory nature through which it was developed. Receiving feedback from stakeholders (assisted living residents and staff in various positions) ensured the intervention strategies were relevant and feasible before the pilot trial. The use of activPAL™ inclinometers reduced the risk of recall bias while including a self-report questionnaire allowed for more detailed assessment of behaviour changes, including domain-specific sitting time. The study was conducted in a relatively short period of time when weather was stable; therefore, any observed changes are not likely explained by a seasonal change in outdoor activity.

Limitations include the low response rate and small sample size, which reduces generalizability of our findings. Due to the small sample size at each site, the study was under-powered, and results of the pilot trial should be viewed with caution. Although the results suggest that the intervention was more successful at Site A than Site B, a larger sample would allow for a better understanding of these differences and what factors contributed to the results. Six weeks is likely too short to see significant changes in health outcomes and a longer intervention and follow up period is needed for future trials. Although the intervention was conceptualized to include staff involvement, there was little day-to-day staff involvement once the intervention was implemented, which also may have reduced the impact of the intervention. Moving forward, larger and longer trials across a variety of assisted living residences are needed to understand the acceptability and effectiveness of SWYC. Furthermore, the development process for SWYC was predominantly influenced by perspectives and feedback from female residents and female staff. Incorporating more men in future studies will help to determine if there are gender differences that should be considered when implementing SWYC. This will also allow for further exploration into why the intervention may be more effective at some sites than others.

## Conclusion

Given the increasing demand for residential care, there is a need for feasible, effective, and affordable strategies to help maintain function among older adults as they transition to assisted living. Clearly now is the time for researchers, knowledge users, and older adults to collaborate on strategies that will promote healthy aging and quality of life. SWYC is a novel, multi-level intervention to reduce sedentary time in assisted living residences. This study provides preliminary evidence of feasibility and effectiveness of the intervention; a larger, randomized controlled trial is warranted.

## Supplementary information

**Additional file 1. Table S1. **Stand When You Can Intervention Strategies.

**Additional file 2.** **Table S2.** Changes in Self-Reported Sedentary Time by Domain.

** Additional file 3. Table S3.** Pre- and Post-Intervention Device-measured Movement Variables, By Weekday and Weekend day.

## Data Availability

The data presented in this paper are available from the corresponding author on reasonable request.

## Abbreviations

ST: Sedentary time
ALRs: Assisted living residences
SWYC: Stand When You Can
AC(s): Activity Coordinator(s)
MVPA: moderate-to-vigorous physical activity
CSEP: Canadian Society for Exercise Physiology
SPPB: Short Physical Performance Battery
LASA: Longitudinal Aging Study Amsterdam
ICECAP – O: ICEpop CAPability Measure for Older People
