# Supplementary material for: Stand When You Can: development and pilot testing of an intervention to reduce sedentary time in assisted living
Source: BMC Geriatr. 2020 Aug 6;20:277. doi: 10.1186/s12877-020-01647-z (PMC7409493; doi:10.1186/s12877-020-01647-z)
Supplement: Supplementary file 1 — Additional file 1. Table S1. Stand When You Can Intervention Strategies. [file 12877_2020_1647_MOESM1_ESM.docx]

Stand When You Can Intervention Strategies

| **Level of the Social Ecological Model** | **Intervention Strategy** | **Description** |
| --- | --- | --- |
| Organization/Policy | Staff Education* | Provide staff with the educational materials that are provided to the residents. (In future, this should be tailored to the staff role(s) and include a formal education session similar to the one provided to residents. It should focus on what staff can do within their role to reduce or interrupt prolonged sedentary time among the residents). |
|  | Standing Breaks in Group Activities* | Encourage Activity Coordinators to incorporate standing breaks in all seated programs or activities. For example, incorporating a card tournament into the weekly routine instead of a regular card game hour would promote more movement between tables. A buffet style meal can be used instead of regular table service. |
|  | Stand When You Can Ambassador | Identify a “SWYC ambassador” each week. This individual is responsible for reminding residents to stand up or break up their sitting time. This ambassador can also assist with sit to stand transitions for the residents as needed. |
|  | Volunteer Opportunities for Residents | Provide volunteer opportunities for residents to complete activities of daily living that they enjoy. This can include things like helping set up or take down from activities or assisting with other chores (i.e. gardening, helping to refill salt and pepper shakers, etc.) |
| Physical Environment | Point of Decision Prompts* | Create and place signs suggesting residents “Stand and Chat” in the common areas and at the standing table. Place a sign reminding residents to stand during commercial breaks in the TV lounge. |
|  | Standing Table in Common Area* | Place a sturdy, bar height table in the common area of the lounge. The table can provide residents with an alternative to sitting while they are socializing or having tea/coffee. A point-of-decision prompt can be placed at the table to encourage use. |
| Social Environment | Weekend Scavenger Hunt* | Implement a weekend scavenger hunt to address the lack of organized activities on weekends. E.g. In a cribbage-themed scavenger hunt, residents are given a score card to record which cards form their cribbage hand. Cards are placed around the common areas (i.e. lounge, hallways, etc.) of the residence on Friday afternoon. Residents search for the cards over the weekend and hand in their score card on Monday; the winning hand receives a prize. In the pilot trial, all residents that completed the scavenger hunt were entered into a draw for a $5 gift card. |
|  | Buddy System | A resident-resident buddy system will pair up residents as accountability buddies. These partnerships will focus on providing companionship and support for sustaining behaviour change related to reducing or breaking up prolonged sedentary time. A staff-resident buddy system will pair a staff member with a resident(s) to help facilitate walks or standing breaks around the staff member’s shift. |
|  | Family Support | Educational materials are provided to family members of all residents. Family members can also be invited to the formal education session and encouraged to support the resident in breaking up periods of prolonged sitting. |
| Individual Behaviour | Educational Workbook* | Provide residents with an educational workbook including information about sedentary behaviour and examples of sedentary behaviour. The workbook includes health benefits of reducing sedentary behaviour and a Frequently Asked Questions section. |
|  | Education Session* | Provide a 60-minute education session in a group-based environment; for the pilot trial this was facilitated by the researchers. The session encourages brain- storming amongst the residents. The session also covered the workbook, goal setting/barrier identification, increasing awareness of sedentary patterns, and a list of tips and tricks to break-up sedentary time. |
|  | Increased Self Awareness of Sedentary Patterns* | Provide a Sedentary Behaviour Questionnaire for residents to self-assess their own sedentary time. Discussion and reflection is encouraged (i.e. were they surprised? Was the number what they expected?). |
|  | Goal Setting and Barrier Identification* | Educate participants on how to set “SMART” goals to specifically address prolonged sedentary time. This is best done after the self-assessment of sedentary time and an explanation of the strategies sheet. Emphasize breaking up or altering of sitting behaviours, not a complete elimination of sedentary time. Discussion of barriers to achieving these goals can also be addressed during the session. |
|  | “Tips and Tricks for Breaking up Sitting Time” Sheet* | Provide a list of different strategies residents can use to increase standing breaks or light physical activity and to remind them to break up prolonged sedentary time. |

* indicates this strategy was implemented in the SWYC feasibility trial
