# Supplementary material for: Stand When You Can: development and pilot testing of an intervention to reduce sedentary time in assisted living
Source: BMC Geriatr. 2020 Aug 6;20:277. doi: 10.1186/s12877-020-01647-z (PMC7409493; doi:10.1186/s12877-020-01647-z)
Supplement: Supplementary file 2 — Additional file 2. Table S2. Changes in Self-Reported Sedentary Time by Domain. [file 12877_2020_1647_MOESM2_ESM.docx]

Supplemental Table 2. Changes in Self-Reported Sedentary Time by Domain

| **Variable** | **Time point** | **Site A** | | **Site B** | | **Overall** | |
| --- | --- | --- | --- | --- | --- | --- | --- |
|  |  | **Weekday** | **Weekend** | **Weekday** | **Weekend** | **Weekday** | **Weekend** |
| Napping | Pre | 37.50 ± 56.79 | 37.5 ± 56.79 | 45.00 ± 15.00 | 45.00 ± 15.00 | 41.66 ± 36.57 | 41.66 ± 36.57 |
|  | Post | 37.50 ± 56.79 | 60.00 ± 84.85 | 60.00 ± 21.21 | 45.60 ± 20.17 | 50.00 ± 39.69 | 52.00 ± 54.42 |
|  | Cohen’s *d* | 0.00 | 0.31 | 0.82^§^ | 0.03 | 0.22 | 0.23 |
| Reading | Pre | 105.00 ± 133.04 | 52.50 ± 51.23 | 96.00 ± 25.10 | 120.00 ± 42.43^d^ | 100.00 ± 83.52 | 90.00 ± 56.13 |
|  | Post | 120.00 ± 97.98 | 105.00 ± 133.04 | 72.00 ± 50.20 | 36.00 ± 53.67^d^* | 93.34 ± 74.16 | 66.67 ± 96.95 |
|  | Cohen’s *d* | 0.13 | 0.52^¥^ | 0.60 | 1.74^¤^ | 0.08 | 0.29 |
| Listening to Music | Pre | 120.00 ± 109.54 | 45.00 ± 90.00 | 60.00 ± 84.85 | 24.00 ± 32.86 | 86.66 ± 95.39^c^ | 33.34 ± 60.83 |
|  | Post | 30.00 ± 60.00 | 15.00 ± 30.00 | 24.00 ± 53.67 | 24.00 ± 53.67 | 26.66 ± 52.91^c‡^ | 20.00 ± 42.42 |
|  | Cohen’s *d* | 0.01 | 0.45 | 0.51 | 0.00 | 0.78^¥^ | 0.26 |
| Watching TV | Pre | 135.00 ± 102.47 | 157.50 ± 66.52 | 204.00 ± 100.40 | 168.00 ± 130.08 | 173.33 ± 101.49 | 163.33 ± 100.75 |
|  | Post | 135.00 ± 133.04 | 52.50 ± 51.23 | 192.00 ± 50.20 | 192.00 ± 98.59 | 166.67 ± 93.80 | 130.00 ± 106.07 |
|  | Cohen’s *d* | 0.00 | 1.77 | 0.74 | 0.21 | 0.07 | 0.32 |
| Seated Hobbies | Pre | 135.00 ± 75.50 | 120.00 ± 84.85 | 96.00 ± 90.99 | 48.00 ± 78.23 | 113.33 ± 81.85 | 80.00 ± 84.85 |
|  | Post | 75.00 ± 75.50 | 120.00 ± 146.97 | 72.00 ± 98.60 | 48.00 ± 7823 | 73.33 ± 83.67 | 80.00 ± 112.25 |
|  | Cohen’s *d* | 0.79^§^ | 0.00 | 0.25 | 0.00 | 0.49^¥^ | 0.00 |
| Talking to Friends | Pre | 83.75 ± 106.57 | 23.75 ± 27.50 | 126.00 ± 95.8 | 132.00 ± 78.23 | 107.22 ± 96.67 | 83.89 ± 81.23 |
|  | Post | 82.50 ± 66.52 | 62.55 ± 82.57 | 60.00 ± 60.00 | 48.00 ± 54.50 | 70.00 ± 60.00 | 54.47 ± 64.04 |
|  | Cohen’s *d* | 0.13 | 0.62 | 0.82^§^ | 1.24 | 0.46 | 0.40 |
| Computer Use | Pre | 150.00 ± 142.83 | 105.00 ± 102.47 | 144.00 ± 124.42 | 96.00 ± 109.00 | 146.66 ± 124.10 | 100.00 ± 99.50 |
|  | Post | 105.00 ± 141.77 | 123.75 ± 143.03 | 96.00 ± 57.71 | 78.00 ± 78.23 | 100.00 ± 96.05 | 98.33 ± 106.36 |
|  | Cohen’s *d* | 0.32 | 0.15 | 0.50^¥^ | 0.19 | 0.42 | 0.02 |
| Administrative Tasks | Pre | 105.00 ± 102.47 | 67.50 ± 78.90 | 60.00 ± 84.85 | 6.00 ± 13.42 | 80.00 ± 90.00 | 33.34 ± 58.94 |
|  | Post | 45.00 ± 57.45 | 107.55 ± 86.09 | 42.00 ± 58.48 | 48.00 ± 78.23 | 43.33 ± 54.31 | 74.47 ± 82.61 |
|  | Cohen’s *d* | 0.72^¥^ | 0.48^¥^ | 0.25 | 0.75 | 0.49^¥^ | 0.62 |
| Transportation | Pre | 30.00 ± 34.64 | 30.00 ± 34.64 | 24.00 ± 25.10 | 27.60 ± 21.88 | 26.66 ± 27.84 | 28.67 ± 26.29 |
|  | Post | 52.50 ± 51.23 | 34.95 ± 57.46 | 42.00 ± 40.25 | 15.00 ± 15.00 | 46.67 ± 42.72 | 23.87 ± 38.22 |
|  | Cohen’s *d* | 0.52^¥^ | 0.10 | 0.54^¥^ | 0.68 | 0.57^¥^ | 0.15 |
| Church | Pre | 30.00 ± 34.64 | 15.00 ± 30.00 | 36.00 ± 53.67 | 36.00 ± 49.30 | 33.34 ± 43.58 | 26.66 ± 40.92 |
|  | Post | 15.00 ± 30.00 | 30.00 ± 34.64 | 57.00 ± 65.72 | 54.00 ± 61.48 | 38.33 ± 54.66 | 43.33 ± 50.00 |
|  | Cohen’s *d* | 0.46 | 0.46 | 0.35 | 0.32 | 0.10 | 0.37 |

All values expressed as minutes per day (means ± standard deviations); ^‡^ indicates a trend towards significance pre-post intervention; * p< 0.05; **p<0.001; superscript letter indicates values that are trending or significantly different from each other; ^¥^moderate effect size; ^§^large effect size; ^¤^very large effect size
