# Supplementary material for: Stand When You Can: development and pilot testing of an intervention to reduce sedentary time in assisted living
Source: BMC Geriatr. 2020 Aug 6;20:277. doi: 10.1186/s12877-020-01647-z (PMC7409493; doi:10.1186/s12877-020-01647-z)
Supplement: Supplementary file 3 — Additional file 3. Table S3. Pre- and Post-Intervention Device-measured Movement Variables, By Weekday and Weekend day. [file 12877_2020_1647_MOESM3_ESM.docx]

Supplemental Table 3. Pre- and Post-Intervention Device-measured Movement Variables, By Weekday and Weekend day.

| **Variable** | **Time** | **Site A** | | **Site B** | | **Overall** | |
| --- | --- | --- | --- | --- | --- | --- | --- |
|  |  | **Weekday** | **Weekend** | **Weekday** | **Weekend** | **Weekday** | **Weekend** |
| Sitting Time | Pre | 624.14 ± 142.30 | 582.45 ± 132.39 | 585.55 ± 224.71 | 564.30 ± 260.05 | 604.85 ± 178.48 | 573.37 ± 194.78 |
|  | Post | 587.44 ± 119.33 | 562.49 ± 226.27 | 596.93 ± 223.68 | 610.43 ± 247.73 | 592.18 ± 169.09 | 586.46 ± 225.10 |
|  | Cohen’s *d* | 0.28 | 0.11 | 0.05 | 0.18 | 0.07 | 0.06 |
| Stepping Time | Pre | 70.76 ± 49.23 | 65.81 ± 58.63^a^ | 63.24 ± 20.60 | 64.05 ± 17.05^d^ | 67.00 ± 35.80 | 64.93 ± 40.72 |
|  | Post | 98.65 ± 91.26 | 84.68 ± 75.31^a‡^ | 60.47 ± 16.9 | 52.41 ± 11.42^d‡^ | 79.56 ± 65.07^g^ | 68.54 ± 53.55^g^* |
|  | Cohen’s *d* | 0.38 | 0.28 | 0.06 | 0.80^§^ | 0.24 | 0.08 |
| Standing Time | Pre | 185.10 ± 72.41 | 181.45 ± 102.28 | 241.76 ± 158.45 | 242.85 ± 173.75 | 213.43 ± 119.92 | 212.15 ± 138.25 |
|  | Post | 194.61 ± 71.20 | 202.78 ± 70.60 | 207.47 ± 116.94 | 207.17 ± 102.20 | 201.04 ± 91.52 | 204.98 ± 82.84 |
|  | Cohen’s *d* | 0.13 | 0.24 | 0.25 | 0.25 | 0.12 | 0.06 |
| Upright Time | Pre | 255.97 ± 107.49 | 247.30 ± 157.66 | 304.40 ± 160.30 | 306.90 ± 170.70 | 280.13 ± 131.19 | 277.08 ± 158.07 |
|  | Post | 293.27 ± 148.17 | 287.46 ± 137.55 | 267.87 ± 117.34 | 259.58 ±107.02 | 280.58 ± 126.71 | 273.52 ± 117.11 |
|  | Cohen’s *d* | 0.29 | 0.27 | 0.26 | 0.33 | 0.00 | 0.03 |
| Sitting Time in Bouts >30min | Pre | 359.49 ± 138.62 | 373.85 ± 165.49 | 384.70 ± 205.30 | 393.75 ± 203.22^e^ | 372.10 ± 165.67 | 383.80 ± 175.03 |
|  | Post | 324.59 ± 166.39 | 314.11 ± 239.53 | 393.85 ± 179.01 | 444.13 ± 207.22^e‡^ | 359.22 ± 166.97 | 379.12 ± 221.99 |
|  | Cohen’s *d* | 0.23 | 0.29 | 0.05 | 0.25 | 0.08 | 0.02 |
| Steps per Day | Pre | 5769.87 ± 5259.08 | 5294.60 ± 6064.93 | 4559.7 ± 1390.81 | 4619.60 ± 1156.63 | 5164.38 ± 3682.29 | 4957.10 ± 4131.38 |
|  | Post | 8447.60 ± 9878.87 | 6929.40 ± 7772.52 | 4335.33 ± 1002.85 | 3707.40 ± 735.57 | 6391.47 ± 6965.54^h^ | 5318.40 ± 5474.85^h‡^ |
|  | Cohen’s *d* | 0.31 | 0.23 | 0.18 | 0.94^§^ | 0.22 | 0.07 |
| Sit to Stand Transitions per Day | Pre | 51.35 ± 10.76^b,c^ | 44.40 ± 10.04^c^* | 42.87 ± 6.59 | 40.00 ± 10.80 | 47.11 ± 9.53 | 42.20 ± 10.10 |
|  | Post | 57.60 ± 7.99^b^* | 50.80 ± 6.72 | 41.00 ± 5.91^f^ | 37.40 ± 5.97^f^* | 49.30 ± 10.97^i^ | 44.10 ± 9.26^i^** |
|  | Cohen’s *d* | 0.66^¥^ | 0.75^¥^ | 0.30 | 0.30 | 0.41 | 0.29 |

^‡^indicates a trend towards significance pre-post intervention; * p< 0.05; **p<0.001; superscript letter indicates values that are trending or significantly different from each other; ^¥^moderate effect size; ^§^large effect size
